# Proteolysis inhibition by hibernating bear serum leads to increased protein content in human muscle cells

**DOI:** 10.1038/s41598-018-23891-5

**Published:** 2018-04-03

**Authors:** Stéphanie Chanon, Blandine Chazarin, Benoit Toubhans, Christine Durand, Isabelle Chery, Maud Robert, Aurélie Vieille-Marchiset, Jon E. Swenson, Andreas Zedrosser, Alina L. Evans, Sven Brunberg, Jon M. Arnemo, Guillemette Gauquelin-Koch, Kenneth B. Storey, Chantal Simon, Stéphane Blanc, Fabrice Bertile, Etienne Lefai

**Affiliations:** 1 CarMeN Laboratory, INSERM, INRA, University of Lyon, Pierre-Benite, France; 2 Université de Strasbourg, CNRS, IPHC UMR 7178, F-67000 Strasbourg, France; 3 Laboratoire de Spectrométrie de Masse Bio-Organique, 25 rue Becquerel, F-67087 Strasbourg, France; 4 Centre National d’Etudes Spatiales, CNES, 75039 Paris, France; 5 Département Ecologie, Physiologie et Ethologie, 23 rue Becquerel, F-67087 Strasbourg, France; 6 Department of digestive and bariatric surgery, Obesity Integrated Center, University Hospital of Edouard Herriot, Hospices Civils de Lyon, Lyon 1 University, Lyon, France; 7 Faculty of Environmental Sciences and Natural Resource Management, Norwegian University of Life Sciences, 1432, Ås, Norway; 8 Norwegian Institute for Nature Research, 7485 Trondheim, Norway; 9 Department of Natural Sciences and Environmental Health, University College of Southeast Norway, N3800 Bø in Telemark, Bø, Norway; 10 Institute of Wildlife Biology and Game Management, University of Natural Resources and Life Sciences, Vienna, Gregor Mendel Str. 33, A-1180 Vienna, Austria; 11 Department of Forestry and Wildlife Management, Inland Norway University of Applied Sciences, NO-2480 Koppang, Norway; 12 Department of Wildlife, Fish, and Environmental Studies, Swedish University of Agricultural Sciences, SE-901 83 Umeå, Sweden; 13 Institute of Biochemistry and Department of Biology, Carleton University, 1125 Colonel By Drive, Ottawa, ON K1S 5B6 Canada

## Abstract

Muscle atrophy is one of the main characteristics of human ageing and physical inactivity, with resulting adverse health outcomes. To date, there are still no efficient therapeutic strategies for its prevention and/or treatment. However, during hibernation, bears exhibit a unique ability for preserving muscle in conditions where muscle atrophy would be expected in humans. Therefore, our objective was to determine whether there are components of bear serum which can control protein balance in human muscles. In this study, we exposed cultured human differentiated muscle cells to bear serum collected during winter and summer periods, and measured the impact on cell protein content and turnover. In addition, we explored the signalling pathways that control rates of protein synthesis and degradation. We show that the protein turnover of human myotubes is reduced when incubated with winter bear serum, with a dramatic inhibition of proteolysis involving both proteasomal and lysosomal systems, and resulting in an increase in muscle cell protein content. By modulating intracellular signalling pathways and inducing a protein sparing phenotype in human muscle cells, winter bear serum therefore holds potential for developing new tools to fight human muscle atrophy and related metabolic disorders.

## Introduction

Muscle disuse atrophy is common in humans during immobilization, bedrest, spaceflight, denervation, cancer, and ageing, and therefore represents a major health issue^1–3^, and is also observed in a number of non-human models used for atrophy studies^4^. Basic knowledge regarding the underlying mechanisms is continuously growing, and an intricate network of signalling pathways appears to be involved in the regulation of muscle fibre size, including IGF1-AKT-FOXO, inflammatory cytokines, NFκB signaling, myostatin, and glucocorticoids^5^. However, there is still no fully effective therapy or prevention for disuse atrophy.

In humans, bed-rest and inactivity result in the loss of up to 30–40% of muscle volume after 2–4 months of disuse^6–8^, and this loss of muscle strength reaches 54% after 3 months of disuse^6^. Physical inactivity and associated loss of muscle mass and strength have severe deleterious consequences for metabolism and health, inducing metabolic inflexibility that could develop into a reduction in the capacity to oxidize lipid fuels, insulin resistance, and ectopic storage of fat^9^. In contrast, minimal or no loss of skeletal muscle mass and strength has been observed in several species during hibernation, despite prolonged disuse and fasting. Among them, bears (family Ursidae) are quite spectacular in this regard. They remain inactive up to seven months during the denning period, without eating, drinking, urinating, or defecating, and without arousal episodes^10–12^. Yet, a very low loss in protein content (from 4% to 17%) is reported in early winter, and this value remains uniquely stable during the following 3–4 months, whereas muscle and fibre cross-sectional area is preserved^13–15^, and muscle strength only decreases by 23%^15^.

The underlying protein sparing mechanisms in bears have yet to be discovered. So far, the current hypotheses, including recycling of nitrogen^11,16–18^ and existence of a specific antioxidant strategy, still require deeper examination. Strikingly, bear skeletal muscles are resistant to the atrophic effects of denervation in winter, but not in summer^19^, suggesting that the nervous system is not directly involved. The direct corollary is the possible role of still unknown humoral factors in controlling bear muscle adaptive responses to hibernation. In support of this view, winter bear plasma has been reported to induce a 40% decrease in the net proteolytic rate in isolated rat muscles^20^, indicating the presence of circulating factors possibly able to trigger protein sparing during hibernation.

In this study, we exposed cultured human differentiated muscle cells to bear serum collected in winter and summer periods (WBS and SBS, respectively), and measured the impact on cell protein content and turnover. In addition, we explored the signalling pathways that control rates of protein synthesis and degradation. Our results highlight how serum from hibernating bears is able to trigger trans-species effects, hence being a potential source for new therapeutic molecules to fight human muscle atrophy and associated metabolic disorders.

## Materials and Methods

### Bear sample collection

Free-ranging sub-adult (2- to 3-year-old) brown bears (*Ursus arctos*; 16 females and 6 males) were captured during both their active (summer) and inactive (winter) periods in Dalarna and Gävleborg counties, Sweden (see Table 1 for bear characteristics). The same bears were immobilized during winter (February) and recaptured during summer (June), as described previously^21,22^. Blood samples were collected from the jugular vein in tubes containing a clot activator (VenosafeTM VF-109SP, Terumo) within 20 min after darting, then centrifuged (2000g, 10 min) within 1 hour after sampling, and serum was immediately frozen on dry ice until storage at −80 °C. The study was approved by the Swedish Ethical Committee on Animal Experiment (applications #C212/9, #C47/9, #C7/12, #C268/12, and #C18/15), the Swedish Environmental Protection Agency (NV-0758-14), and the Swedish Board of Agriculture (31- 11102/12). All procedures complied with Swedish laws and regulations.

**Table 1:** Characteristics of the brown bears used in this study.

Year of collection	Id Number	Age (years)	Gender	Winter weight (kg)	Summer weight (kg)	Mixes
2011	W1015	2	M	25	27	M1W & M1S
2012	W1017	3	F	56	55	
	W1105	2	F	31.5	na	
	W1104	2	F	30.2	29	
	W1110	2	F	27.3	29	
2013	W1105	3	F	55	60	M2W & M2S
	W1207	2	M	54	64.5	
	W1110	3	F	53	58	
	W1104	3	F	52	57	
	W1202	3	F	48	48	
	W1204	2	M	40	38	
	W1209	2	F	30	27	
2014	W1303	2	F	36	43	M3W & M3S
	W1317	2	M	33	40	
	W1305	2	F	37	49	
	W1303	2	F	36	43	
	W1317	2	M	33	40	
2016	W1404	3	M	50	68	M4W & M4S
	W1407	3	F	74	83.6	
	W1509	2	F	25	37.8	
	W1511	2	F	29	41	
	W1512	2	F	36	51.2	
	N = 22	2.3 ± 0.1	16 F/6 M	40.1 ± 2.8	46.7 ± 3.2	

A total of 22 pairs of bear serum (winter and summer) were collected during the indicated years. Equal volumes of serum were used to obtain four different mixes (M1W to M4W) of winter bear serum (WBS) and four paired mixes (M1S to M4S) of summer bear serum (SBS). All experiments presented in this study were performed with each of the mixes as replicates. Averages are given as mean ± SEM.

We prepared different mixes of winter bear serum (WBS) and of summer bear serum (SBS) using the individual bear samples (Table 1). It is important to note that serum samples were collected from the same animals during both seasons, and that both summer and winter mixes were obtained by pooling the exact same volume of serum for all bears. Detailed characteristics of the sera in terms of metabolite and hormone levels and more global proteomic compositions have been published previously from the same bear population^23,24^.

### Culture of human skeletal muscle cells and treatments with bear serum mixes

Human muscle cells were derived from *vastus lateralis* muscle biopsies obtained from healthy control donors (Diomede experimental protocol). All procedures were approved by the French Ethical Committee SUD EST IV (Agreement #12/111 A 13-02) and performed according to the French legislation (Huriet’s law). All patients gave their written consent after being informed of the nature, purpose, and possible risks of the study. The myoblasts were purified and differentiated into myotubes according to the procedure previously described in detail^25^. After five days of differentiation in the standard differentiation medium (DMEM medium), containing glucose (1 g/l) and fetal bovine serum (FBS, 2%), myotubes were washed with phosphate-buffered saline (PBS) and then incubated at 37 °C with 5% of either SBS or WBS. The 5% concentration of bear serum was chosen on the basis of the previous work from Fuster *et al*.^20^, who tested the effects of plasma on *ex vivo* rat muscles. A control group was set as myotubes kept in differentiation medium containing 2% fetal bovine serum (FBS condition). For all experiments, repetitions were performed on cell preparations coming from different donors.

### Myosin heavy chain imaging and cell surface determination

After exposure to FBS, SBS or WBS (48 h), human muscle cells were washed twice with PBS and fixed with 4% formaldehyde for 10 min, then permeabilized with 0.1% Triton X-100/PBS for 15 min and blocked by 1% bovine serum albumin for 30 min at room temperature. Myosin heavy chain protein of human myotubes was detected with primary antibody MF-20, followed by a secondary antibody Alexa Fluor® 555 Anti-Mouse IgG (H + L) incubation. Then, the area occupied by myotubes was measured, as described in detail previously^26^. For each well, corresponding to one mix applied to one cell preparation, at least ten pictures were taken to determine the percentage of surface occupied by myotubes.

### Measurement of protein degradation and synthesis rates

Protein degradation rates were assayed, as previously described^26^. Human muscle cells were first incubated for 24 hours with [^3^H]-L-Tyrosine (2 μCi/ml), then washed three times with PBS, and shifted in chase medium (containing nonradiolabeled tyrosine) for two hours. The release of TCA-soluble radioactivity in the culture medium was assayed for the 6 hours following 24 hours of incubation in SBS and WBS conditions to determine the rate per hour of free tyrosine release. The total amount of incorporated radioactivity was then determined in the whole cell culture to calculate the degradation rate relative to the amount of labeled protein, expressed in percentage per hour. Protein degradation rates were also measured in the presence of proteasome (Bortezomib/PS-341, 1 μM) and lysosome (Concanamycin A, 0.1 μM) inhibitors, as previously described^27^. Briefly, after cell protein labeling (24 hours, see above), inhibitors or DMSO were added to FBS, SBS and WBS conditions one hour before monitoring the release of TCA-soluble radioactivity for 6 hours. All procedures were repeated with three different primary myotube preparations.

Protein synthesis rates were assessed using two different procedures. Incorporation of radiolabeled tyrosine was monitored, as already described^26^. Briefly, myotubes were exposed to SBS and WBS for 24 or 48 hours, and [^3^H]-L-Tyrosine (2 μCi/ml) was then added to the medium. After 2 hours incubation, cells were scraped in 10% TCA to precipitate proteins. The pellet was resuspended in lysis buffer (Tris-HCl 20 mM, NaCl 138 mM, KCl 2.7 mM, MgCl_2_ 1 mM, Glycerol 5%, NP 40 1%, EDTA 5 mM, Na_3_VO_4_ 1 mM, NaF 20 mM and DTT 1 mM), and protein concentration and radioactivity were quantified. Protein synthesis rates are presented as means ± SEM of at least three determinations with different myotube preparations. We also used puromycin incorporation assays to determine protein synthesis rates using the SunSET method^28^. Cells were exposed to SBS and WBS for 24 or 48 hours and puromycin (5 μM) was added for the last 30 minutes. Measurements for the FBS condition were only performed after 48 hours. Cells were then scraped in lysis buffer to extract proteins. After quantification, 20 μg of proteins were immunoblotted using an anti-puromycin antibody (see western blot procedures).

### Western blotting

After bear serum incubation, cells were scraped into 200 μl of ice-cold lysis buffer (TRIS-HCl 20 mM, NaCl 138 mM, KCl 2.7 mM, MgCl_2_ 1 mM, Glycerol 5%, NP 40 1%, EDTA 5 mM, Na_3_VO_4_ 1 mM, NaF 20 mM and DTT 1 mM) supplemented with a protease inhibitor cocktail (Sigma-Aldrich, France). Protein concentration was determined by Bradford quantification. Western blotting was performed, as described previously^29^, loading 20 μg of total protein on precast gels (Mini-protean TGX Stain-free^TM^ gel, Biorad, France). After migration, gels were UV exposed for 3 minutes and pictures were taken for further quantification of protein loading. After semi-dry transfer, all membranes were blocked with 4% BSA (Bovine Serum Albumin, Euromedex, Souffelweyersheim, France) before incubation with primary antibodies (see Table S1). Corresponding secondary HRP antibodies were used for chemiluminescence revelation (Chemidoc Bio-Rad).

### Quantification of mRNAs by real-time RT-PCR

Total RNA was isolated using the TRIzol reagent (Invitrogen, Courtaboeuf, France) according to the manufacturer’s instructions. RT-qPCR was performed as previously described^29^. TBP (Tata-box Binding Protein) mRNA levels were determined in each sample and was used as internal standard for normalization. No change in TBP expression was found comparing FBS, SBS and WBS conditions. The primers and real-time PCR assay conditions are listed in Table S2.

### Statistical analysis

All data are presented as means ± SEM, and fold change always related to the SBS condition. To compare the SBS and WBS conditions, statistical significance was determined using paired student t-tests, with * indicating a p value < 0.05, **p < 0.01, and NS not significant.

## Results

### Human muscle cells exposed to WBS exhibit higher protein content

**Figure 1:**
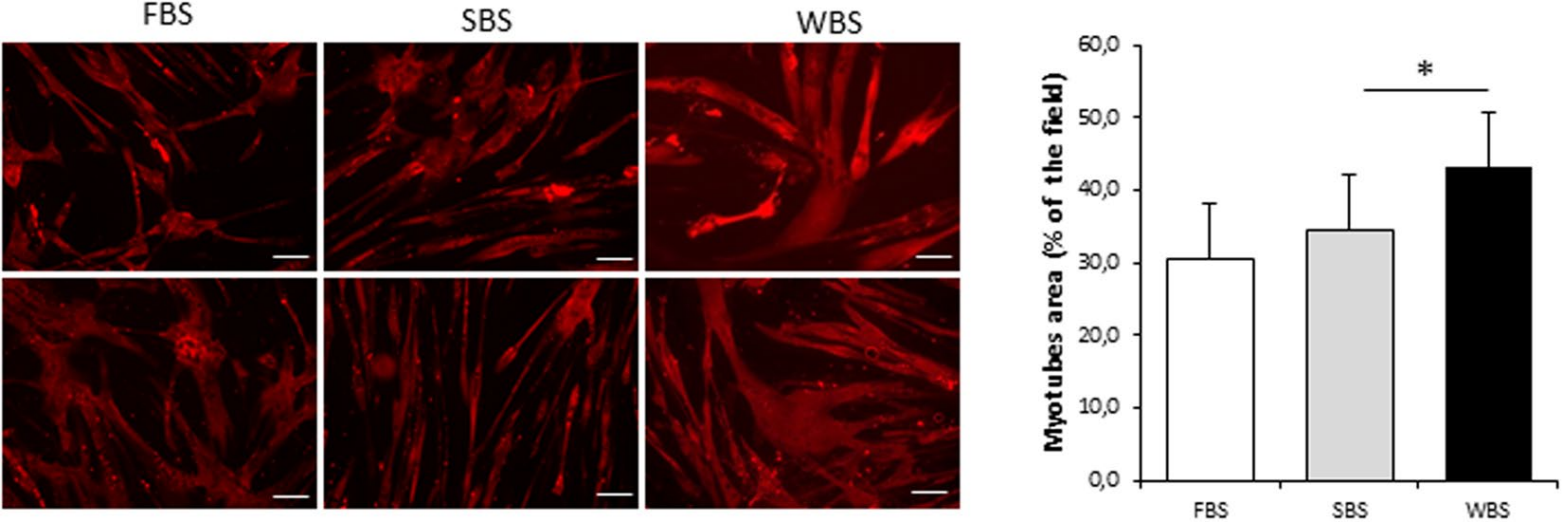
Winter bear serum promotes an increase in human muscle cell size. Illustrative immunodetection and corresponding quantification of myosin heavy chain in cultured myotubes upon standard culture condition with fetal bovine serum (FBS), winter bear serum (WBS) or summer bear serum (SBS) treatment. Results are the mean ± SEM of 6 independent experiments (different cell preparations and bear serum mixes). Scale bar: 100 µm. Significant differences result from paired T-tests (*p < 0.05).

To evaluate the ability of bear serum to impact human muscle cell protein content, we first incubated quiescent differentiated polynucleated myotubes with bear serum collected in winter during hibernation and in summer during their active period. After 48 hours, we determined the cell content of heavy chain myosin by immunofluorescence and quantified myotube surface area (Fig. 1). While no difference could be observed between FBS and SBS conditions, the WBS treatment induced a significant increase in muscle cell surface area (+21.7 ± 7.2% p = 0.015) compared to SBS, indicating higher protein content.

### Reduced protein turnover in human myotubes exposed to WBS compared to SBS

**Figure 2:**
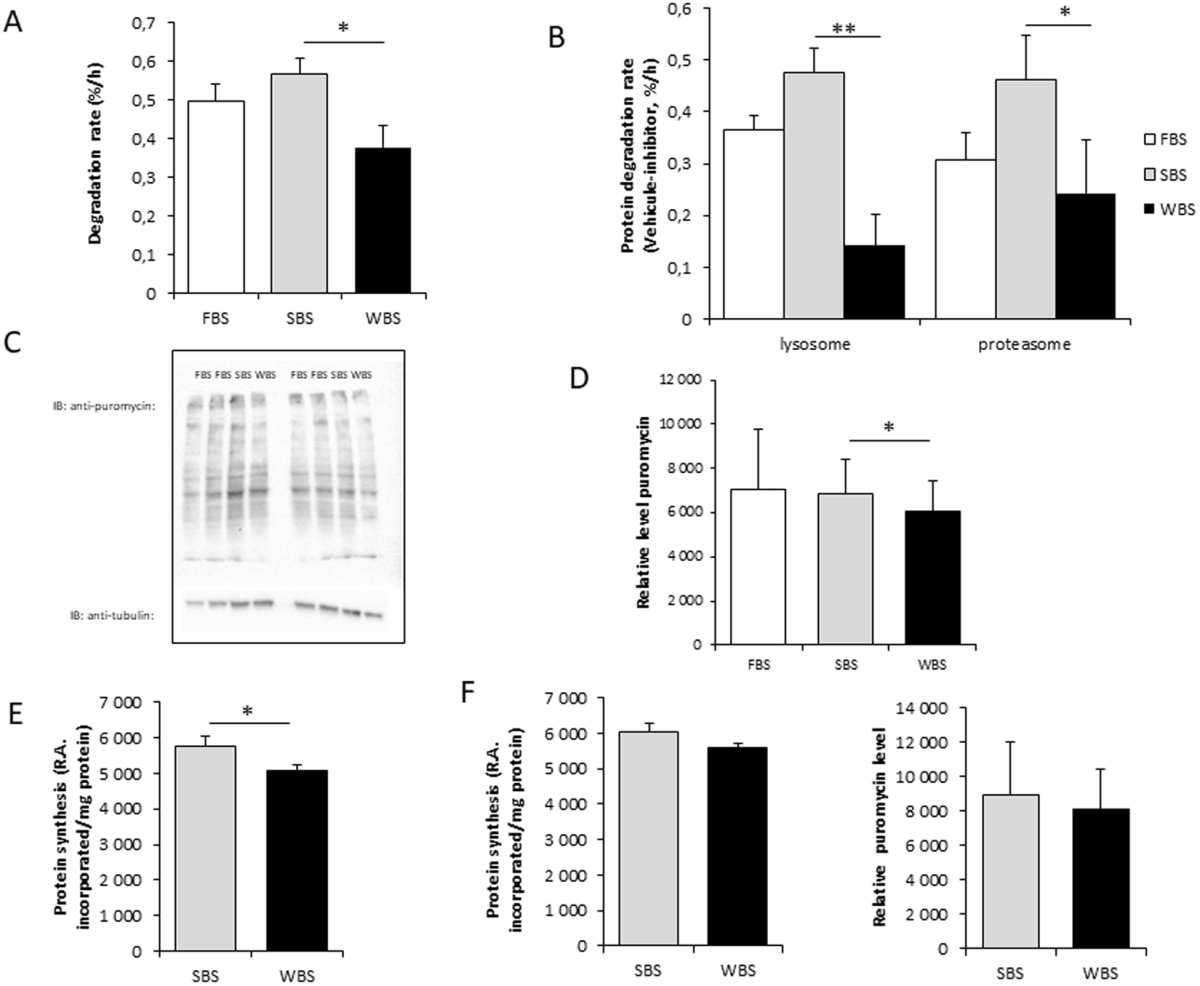
Winter bear serum inhibits protein degradation and synthesis rates in human muscle cells. Overall protein degradation rates in human myotubes exposed for 24 hours to FBS, SBS or WBS (**A**), and rates of proteolysis after proteasomal or lysosomal inhibition (**B**) were measured. For the latter, twenty-four hours after serum exposure, inhibitors (1 µM Bortezomid [PS-341], 0.1 µM concanamycin [Conc. A], or DMSO only [Veh.]) were added, and rates of proteolysis were determined for six hours. Proteasomal and lysosomal degradation rates were expressed as the difference between total and specifically inhibited rates. Overall protein synthesis rates were assessed in human myotubes with quantification of puromycin incorporation after 48 hours of exposure to FBS, SBS or WBS (**C**,**D**). Protein synthesis rates were also assessed by measuring [3H]-tyrosine incorporation in human myotubes exposed for 48 hours to SBS or WBS (**E**). (**F**) Shows incorporation rates of [3H]-tyrosine and puromycin after 24 hours. Results are the mean ± SEM of at least 3 independent experiments measured in duplicate. Significant differences result from paired T-tests (*p < 0.05; **p < 0.01).

To investigate the mechanisms driving the change in protein content of treated human myotubes, we next determined the rates of protein synthesis and degradation. Whereas incubation with SBS did not change the degradation rate compared to standard culture conditions (FBS condition), a 33% lower protein degradation rate (0.56 ± 0.04 vs 0.37 ± 0.06, p = 003) was recorded after 24 hours of incubation with WBS compared to the SBS condition (Fig. 2A). To determine whether this could be related to proteasomal and/or lysosomal pathways, we next measured degradation rates in the presence of proteasome (PS-341, Bortezomid) or lysosomal proton pump (concanamycin A) specific inhibitors. Calculation of the inhibitor-sensitive fraction by subtracting the rates of proteolysis in cells treated with inhibitors from those in untreated cells then represented the actual contribution of each pathway in the total degradation process. As shown in Fig. 2B, the contribution of both proteasomes and lysosomes to the degradation rates were dramatically decreased (−47% and −71%, respectively) in human myotubes exposed to WBS compared to the SBS condition, thus showing the involvement of both systems in the response to WBS.

We next quantified protein synthesis rates in myotubes exposed to bear serum by measuring the incorporation of puromycin (Fig. 2C,D). SBS did not change protein synthesis rates compared to standard culture conditions (FBS condition) whereas the puromycin incorporation was significantly decreased under the WBS condition. The same result was observed when measurements were performed using incorporation of [^3^H]-tyrosine into neosynthesized proteins. A mild but significant decrease was found only after 48 hours of treatment in WBS- compared to SBS- treated cells (Fig. 2E), while after 24 hours of treatment, rates were slightly, but not significantly, lower (Fig. 2F). The human myotubes exposed to WBS thus displayed a mildly reduced rate of protein synthesis, whereas protein degradation was dramatically inhibited.

### Effects of WBS on proteasomal and lysosomal degradation pathways

**Figure 3:**
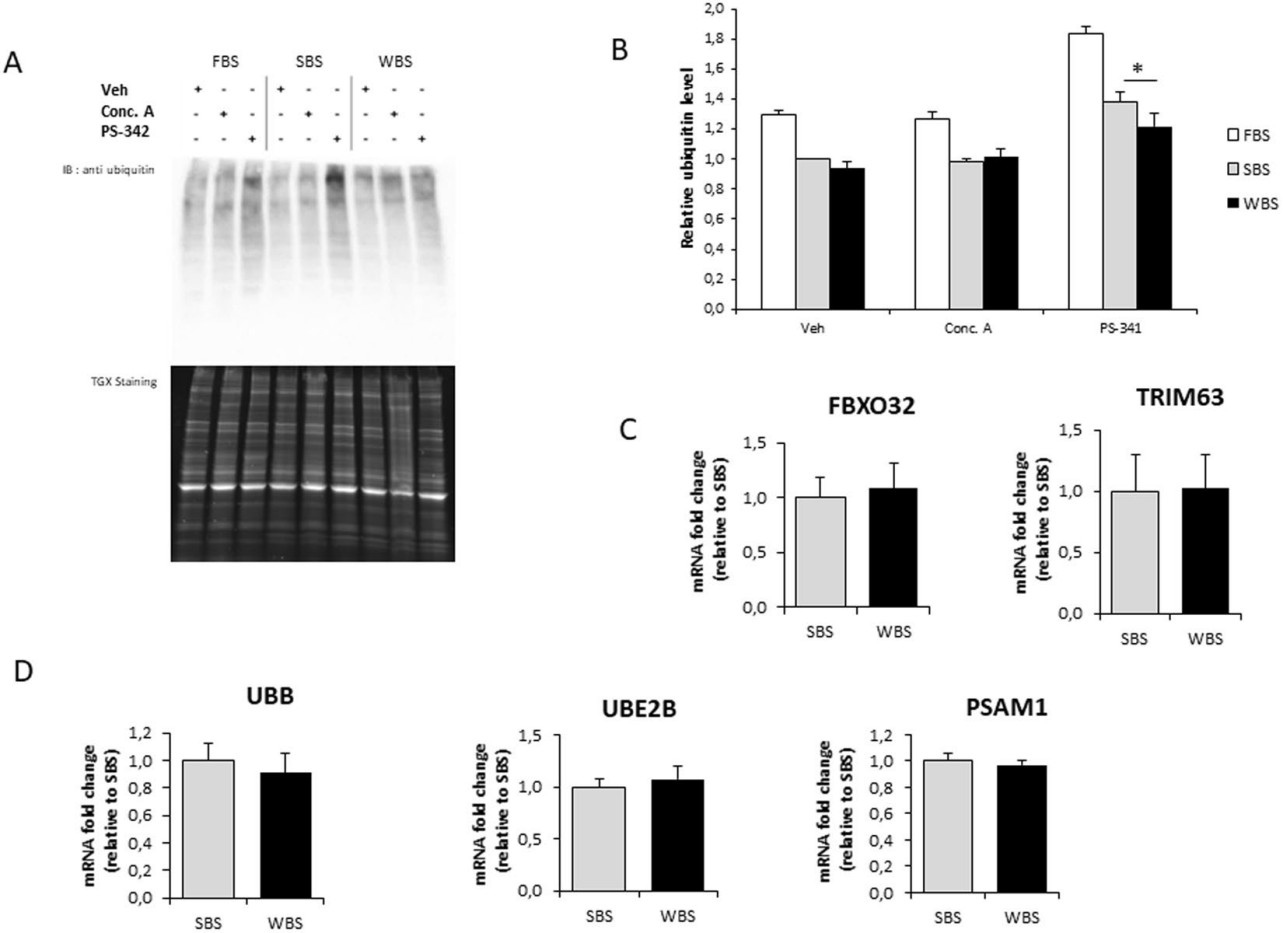
Winter bear serum inhibits proteasomal degradation in human muscle cells. Illustrative immunodetection (**A**) and corresponding quantification (**B**) of ubiquitinated proteins in human myotubes exposed for 24 hours to FBS, SBS or WBS. Quantifications were performed after one hour of proteasomal (1 µM Bortezomid [PS-341]) or lysosomal (0.1 µM concanamycin [Conc. A]) inhibition, and in control conditions (DMSO only [Veh.]). Myotube expression levels of muscle specific E3-specific ligase FBXO32 (MuRF1) and TRIM63 (Atrogin-1) (**C**), and of ubiquitin (UBB) and two components of the proteasome system (UBE2B and PSAM1) were measured by RT-qPCR, normalized against TBP mRNA levels, and expressed as a fold change relative to the SBS condition. Results are the mean ± SEM of at least 3 independent experiments. Significant differences result from paired T-tests (*p < 0.05).

To explore the ubiquitin-proteasome system in myotubes exposed to bear serum, we first quantified the level of ubiquitinated proteins in human myotubes under FBS, SBS and WBS conditions (Fig. 3A). Comparing SBS and WBS conditions, no differences could be found in the control (veh) and lysosomal inhibition (Conc.A) conditions, whereas proteasome inhibition (PS-341) promoted an increase in the amount of ubiquitinated protein (Fig. 3B). In this latter condition, the increase was significantly reduced in the WBS compared to SBS condition, suggesting that WBS alone lowers proteasome activity. The overall process from ubiquitin ligation to protein degradation was explored with the quantification of the mRNA levels of ubiquitin (UBB), E2 Ubiquitin-Conjugating Enzyme B (UBE2B), the proteasome subunit alpha1 (PSMA1), and muscle specific E3 ligases TRIM63 and FBXO32 (Fig. 3C,D). Expression levels of all of these messengers were similar in SBS and WBS conditions, indicating that lower proteasomal degradation due to WBS treatment was unlikely to originate from transcriptional regulations and would most likely be due to lower activities of related components.

**Figure 4:**
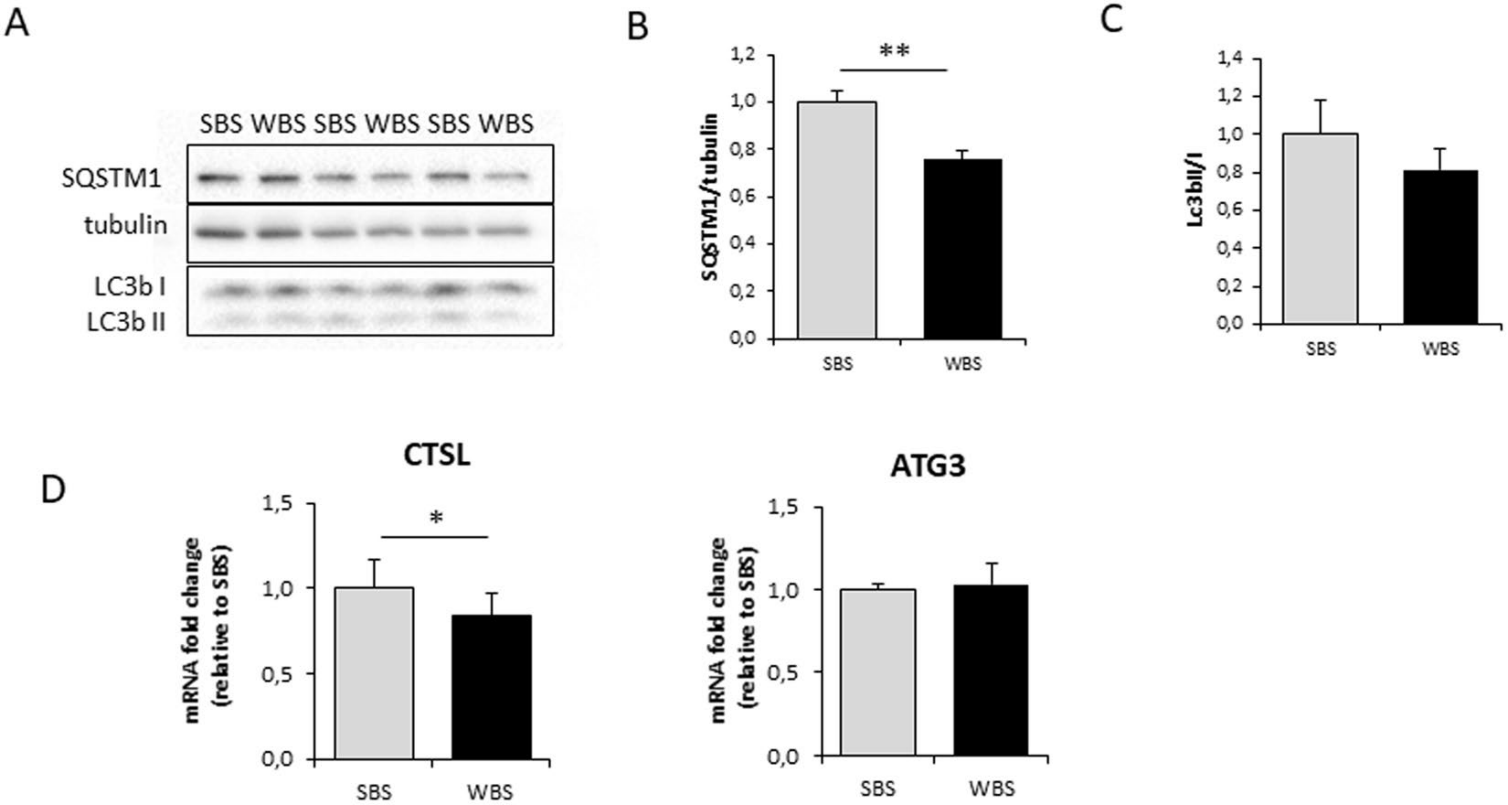
Winter bear serum impacts main factors of autophagy/lysosomal degradation in human muscle cells. Illustrative immunodetection (**A**) and corresponding quantification (**B**) of SQSTM1 (p62) and the LC3b II/I ratio in human myotubes exposed for 24 hours to SBS or WBS (**C**). Myotube expression of CTSL and ATG3 was measured by RT-qPCR, normalized against TBP mRNA levels, and expressed as a fold change relative to the SBS condition (**D**). Data are the mean ± SEM of at least 3 independent experiments. Significant differences result from paired T-tests (*p < 0.05; **p < 0.01).

Autophagy was investigated by quantification of SQSTM1 (p62) protein and LC3II/I ratios (Fig. 4A–C). During induction of the autophagosomal process, SQSTM1 binds the ubiquitinated proteins and LC3I is lipidated to LC3II, resulting in an increase in the LC3II/I ratio. We observed a highly significant lower abundance in SQSTM1 under WBS versus SBS conditions, whereas LC3b II/I ratios were not significantly decreased. At the mRNA level, expression of the E2-conjugating enzyme ATG3, and the lysosomal hydrolase cathepsin L (CTSL) were quantified in WBS- and SBS-treated cells (Fig. 4D). Whereas ATG3 expression was not modified, expression of CTSL was lower in the WBS condition, which would be in line with reduced lysosomal protein degradation.

### Effects of WBS on protein metabolism signaling pathways

**Figure 5:**
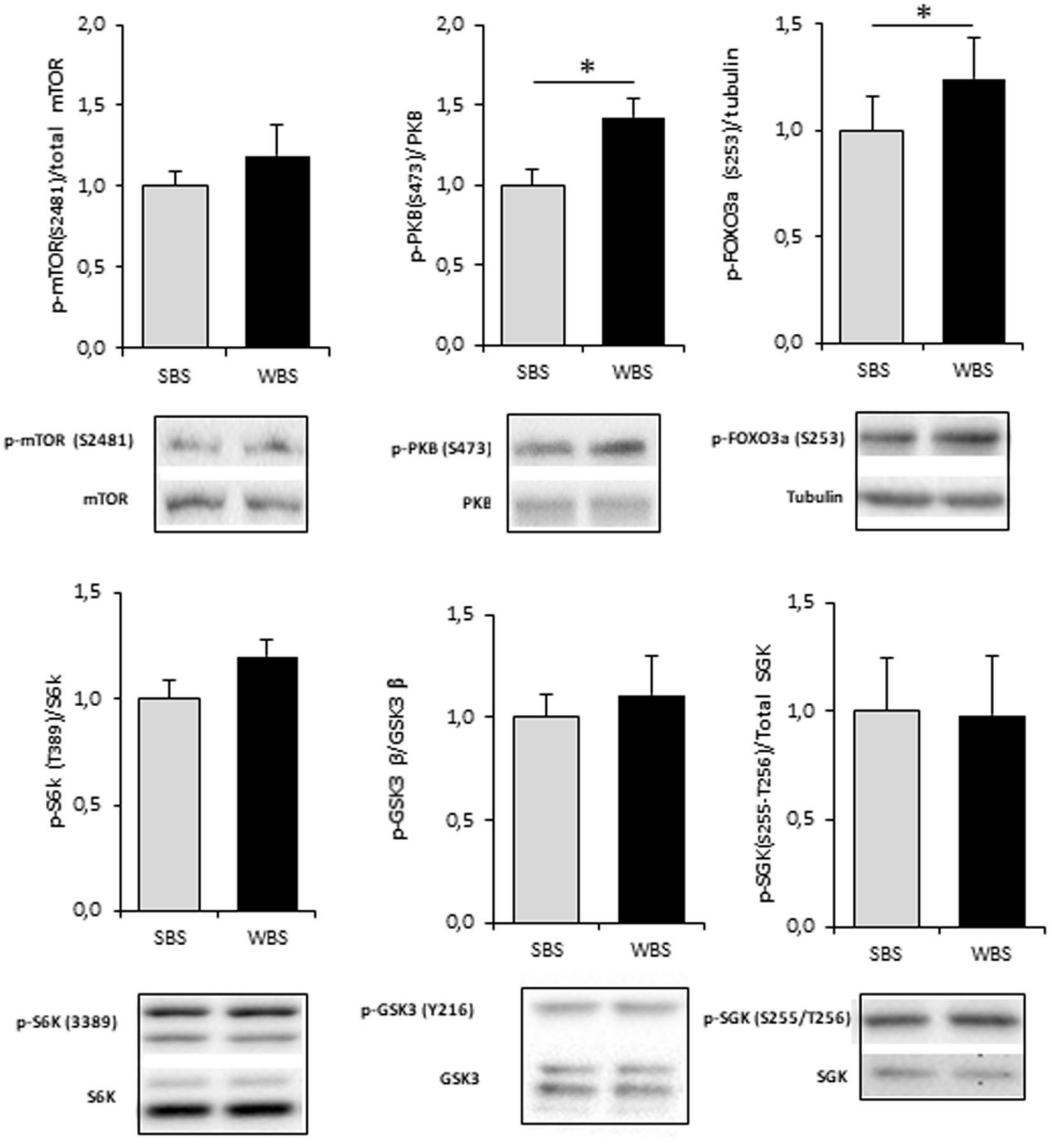
Winter bear serum activates mTOR/PKB pathways in human muscle cells. Illustrative blots and corresponding quantification of mTOR, PKB, FOXO3a, S6K, GSK3beta, SGK and their phosphorylated forms. Results are the mean ± SEM of at least of 3 independent experiments. Significant differences result from paired T-tests (*p < 0.05).

Intracellular signaling pathways were investigated by the quantification of the phosphorylated fraction of several key actors involved in the regulation of protein turnover (Fig. 5). The fraction of phosphorylated PKB (protein kinase B), but not mTOR (mechanistic Target of Rapamycin), was found at higher levels in WBS- than SBS-treated cells. Accordingly, the downstream target FOXO3 (Forkhead box O3) was also found to be hyper-phosphorylated and thus presumably inactivated in the WBS condition. Meanwhile, similar phosphorylation levels of serum/glucocorticoid-induced kinase 1 (SGK1) were recorded in SBS and WBS conditions, indicating that it might not participate in the higher phosphorylation level of FOXO3 that we observed upon WBS exposure. Finally, the levels of phosphorylation of S6K (ribosomal protein S6 kinase) and GSK3beta (glycogen synthase kinase 3ß) were found to be not statistically different between SBS and WBS conditions.

## Discussion

Despite prolonged periods of fasting and inactivity, conditions which induce dramatic muscle wasting in humans^30,31^, the muscle mass and strength of hibernating bears are remarkably well preserved^14,32–34^. This preservation is part of an overall adaptation scheme in which energy and metabolic changes are triggered in the cardiovascular system, kidney, liver, and intestine (microbiota). In this regard, the coordination of tissue and organ functions may involve circulating factors, and winter bear plasma has been reported to decrease the net proteolytic rate in isolated rat muscles^20^. As a new approach to overcome the lack of efficient treatment or prevention of human muscle disuse-induced atrophy, we explored here how WBS can affect protein turnover in human muscle cells, studying protein synthesis and degradation, as well as key actors involved in regulating pathways. To examine if circulating factors could impact human muscle cell physiology, we choose to incubate cells with bear serum rather than plasma that may contain coagulation inhibitors susceptible to induce effects *per se* like alteration of enzyme activities. Application of WBS to human myotubes resulted in a unique situation, where a marked inhibition of proteolysis and a slight decrease in protein synthesis were observed. The resulting positive balance in protein turnover led to a higher protein content in muscle cells.

Muscle protein turnover is a tightly regulated process, the equilibrium between catabolic and anabolic pathways being under the control of several endocrine, paracrine, and autocrine factors (for review see^5,35–38^). In any situation of muscle atrophy, whether it results from immobilization, denervation, fasting, ageing (sarcopenia), inflammation, or cancer (cachexia), muscle protein degradation is activated through two intricate systems: the ubiquitin proteasome and autophagy systems^39,40^. We show here that, in human myotubes, both proteasomal and lysosomal system activities were reduced in the WBS condition compared to SBS. Considering signaling pathways, the canonical PKB actor was found activated and accordingly, the downstream FOXO3 transcription factor was inactivated by higher phosphorylation. Coordinated activation of this signaling pathway may thus explain most of the observed effects on the proteasomal and lysosomal systems, considering their role in the control of protein degradation and regulation of muscle mass^5,27,36,41^.

Inactivation of FOXO3 is consistent with the recent demonstration of FOXO4 inactivation in hibernating squirrels^42^. Although serum/glucocorticoid-induced kinase 1 (SGK1) has been shown to be involved in the prevention of atrophy in hibernating 13-lined ground squirrels^34,43^, we recorded similar phosphorylation levels of SGK1 in myotubes exposed to either SBS and WBS conditions. Thus, in our human muscle cell model, SGK1 may not participate in the regulating the higher phosphorylation level of FOXO3 that we observed upon WBS exposure. The observed increase in PKB phosphorylation of treated myotubes could also reflect the modified insulin sensitivity of the bear skeletal muscle tissue during hibernation^44^ and highlights a promising future in the field of human diabetes.

Among the few factors inhibiting muscle proteolysis described to date, insulin and amino-acids are the best characterized^45–48^. Their circulating levels are increased in the post-prandial situation, at the same time substrates and energy are available for muscle protein metabolism^47,49^. These anabolic conditions trigger activation of the PKB/mTOR pathway, with subsequent inhibition of proteolysis and concomitant activation of proteosynthesis. Strikingly, we found an activation of PKB with positive consequences on protein content in human myotubes exposed to WBS, despite the fact that the serum originated from animals that had been food deprived and physically inactive for at least three months. How WBS can mimic an anabolic situation for human cultured muscle cells remains to be elucidated. Moreover, considering the ability of WBS to reproduce in treated human cells the regulation of protein turnover already described in hibernating bear muscle (i.e. lower synthesis and lower degradation rates^13^), it is highly probable that the maintenance of muscle mass during bear hibernation involves one or several circulating factors.

To explain how WBS can activate PKB, leading to FOXO inactivation and protein degradation inhibition in human muscle cells, active circulating factors may be sought among those whose concentrations are known to change between seasons, either due to the central regulation of hibernation via the hypothalamic-pituitary axis^50,51^, and/or in relation to the nutritional status of the animals through their gut-liver axis^52,53^. Although several differences have already been highlighted in the blood composition of active and hibernating bears^11,23,24,51–57^, no particular factors have yet been identified that specifically control muscle adaptive responses. Serum conveys hormones and growth factors that are major actors in muscle mass regulation^58,59^. However, considering the reduced metabolic rate in hibernating bears^60^, it is difficult to imagine an increase in such factors during winter. Of note, higher levels of blood proteins and hematocrit have been reported during hibernation^23^, which can likely be attributed to dehydration in hibernating bears. Such seasonal changes may lead to higher concentrations of circulating factors during winter, without any change in their production or clearance.

Among the circulating components that are known to show seasonal regulation in bears, fatty acids, whose composition are changed due to prolonged fasting, could play a role. Lipids are known regulators of muscle mass^61^, notably through saturated fatty acids and their ability to generate ceramides. Whereas both lipid classes negatively affect muscle mass, the positive impact of other lipid moieties cannot be excluded. Notably ketone bodies could be involved, since their circulating levels are increased by fasting^62,63^ and hibernation^64^, and they are known to be involved in the regulation of muscle mass^65^. In hibernating bears, fasting is also associated with modifications in urea and nitrogen metabolism^66^. The role of specific amino acids can not be ruled out, such as BCAA^67^ or citrulline^68^, even if supplementation experiments have not yet produced conclusive results. Finally, from the recent proteomic characterization of WBS and SBS^69^, it has been proposed that α2-macroglobulin, a non-specific protease inhibitor that exhibits higher circulating levels in winter bears, could be involved in the reduction in protein degradation in isolated rat muscle when incubated with winter bear plasma^20^. Therefore, α2-macroglobulin could be involved in the reduced proteolysis of human muscle cells that we observed here upon WBS treatment.

In conclusion, the circulating components that induce the potent trans-species effects on human muscle cells and that make their phenotype closely mimic that of winter bear muscles are probably those that are responsible for the maintenance of muscle mass and strength in hibernating bears. Their identification will no doubt pave the way for a new field of studies that will investigate novel solutions to prevent and/or reverse muscle atrophy in humans.

## Electronic supplementary material

Tables S1-S2

Supplementary Information
