# Supplementary material for: Proteolysis inhibition by hibernating bear serum leads to increased protein content in human muscle cells
Source: Sci Rep. 2018 Apr 3;8:5525. doi: 10.1038/s41598-018-23891-5 (PMC5883044; doi:10.1038/s41598-018-23891-5)
Supplement: Supplementary file 1 — Tables S1-S2 [file 41598_2018_23891_MOESM1_ESM.docx]

**Supplementary tables**

**Proteolysis inhibition by hibernating bear serum leads to increased protein content in human muscle cells**

Stéphanie Chanon^1^, Blandine Chazarin^2,3,4^, Benoit Toubhans^1^, Christine Durand^1^, Isabelle Chery^2,5^, Maud Robert^1,6^, Aurélie Vieille-Marchiset^1^, Jon E. Swenson^7,8^, Andreas Zedrosser^9,10^, Alina L. Evans^11^, Sven Brunberg^7^, Jon M. Arnemo^11,12^, Guillemette Gauquelin-Koch^4^, Kenneth B Storey^13^, Chantal Simon^1^, Stéphane Blanc^2,5^, Fabrice Bertile^2,3^, Etienne Lefai*^1^

Please find below supplementary tables S1 and S2.

**Supplementary table S1: list of antibodies**

| Name | Supplier | Reference | Dilution | Species |
| --- | --- | --- | --- | --- |
| Phospho-p70 S6 Kinase (Thr389)(1A5) | Cell signaling | 9206 | 1/500 | Mouse |
| p70 S6 Kinase (49D7) | Cell signaling | 2708 | 1/1000 | Rabbit |
| Phospho-FKHRL1 (Ser253) | Sigma | SAB4504409 | 1/1000 | Rabbit |
| Tubulin | Sigma | T5168 | 1/2000 | Mouse |
| Phospho-SGK (ser255/thr256) | Upstate | 36002 | 1/1000 | Rabbit |
| SGK | Cell signaling | 3272 | 1/1000 | Rabbit |
| Lc3b | Sigma | L7543 | 1/1000 | Rabbit |
| Phospho-PKB (ser473) | Cell signaling | 4060 | 1/1000 | Rabbit |
| PKB | Cell signaling | 4691 | 1/1000 | Rabbit |
| Phospho-mTOR (ser2481) | Cell signaling | 2974 | 1/1000 | Rabbit |
| mTOR | Life technologies | AH01232 | 1/1000 | Mouse |
| GSK3 beta | Cell signaling | CS9315 | 1/2000 | Rabbit |
| Phospho-GSK3 beta (Y216) | Abcam | AB75745 | 1/2000 | Rabbit |
| Anti-SQSTM1 / p62 | Abcam | AB56416 | 1/1000 | Mouse |
| Ubiquitin (P4D1) | Cell signaling | 3936 | 1/1000 | Mouse |
| Puromycin | Merk Millipore | MABE343 | 1/10000 | Mouse |
| Anti-Mouse IgG (H+L)-HRP conjugate | BioRad | 172-1011 | 1/10000 | Goat |
| Anti-Rabbit IgG (H+L)-HRP conjugate | BioRad | 172-1019 | 1/10000 | Goat |
| AntiMyosin Heavy Chain (MF20) | DSHB | MF20c | 1/50 | Mouse |
| Alexa Fluor® 555 Anti-Mouse IgG (H+L), highly cross-adsorbed | Life Technologies | A 21424 | 1/1000 | Goat |

**Supplementary table S2 : List of primers used for RT-qPCR**

| **Gene** | |  | **Primer** |
| --- | --- | --- | --- |
| **Hs.CTSL** | Forward | | 5'-CTGGACTCTGAGGAATCCTA-3' |
|  | Reverse | | 5'-AAGGACTCATGACCTGCATC-3' |
| **Hs.FBXO32** | Forward | | 5'- CGGGAACATCAACATGTGGG-3' |
|  | Reverse | | 5'-GTTGTAGGCACAAAGGCAGG -3' |
| **Hs.TRIM63** | Forward | | 5'-TCCATGTGCAAGGTGTTTGG -3' |
|  | Reverse | | 5'-ACTGTTCTCCTTGGTCACTC -3' |
| **Hs.ATG3** | Forward | | 5'-AGGCTACCCTAGATACAAGG-3' |
|  | Reverse | | 5'-ACCATAATCGTGGAGTCTGG-3' |
| **Hs. UBB** | Forward | | 5'-GAGACGGCGTCTACGTGAGG-3' |
|  | Reverse | | 5'-GCATTTTGACGTGTTAGCGG-3' |
| **Hs.UBE2B** | Forward | | 5'-GCTCATGCGGGATTTCAAGC-3' |
|  | Reverse | | 5'-CTGCATTCCACTGCATGATG-3' |
| **Hs.PSAM1** | Forward | | 5'-CAACACAACGATATGGCCGG-3' |
|  | Reverse | | 5'-TCCAATGGACATGGCTCTGC-3' |
| **Hs. TBP** | Forward | | 5’- AGACCATTGCACTTCGTGCC-3’ |
|  | Reverse | | 5’- CCTGTGCACACCATTTTCCC-3’ |
