# Supplementary material for: Proteolysis inhibition by hibernating bear serum leads to increased protein content in human muscle cells
Source: Sci Rep. 2018 Apr 3;8:5525. doi: 10.1038/s41598-018-23891-5 (PMC5883044; doi:10.1038/s41598-018-23891-5)

Supplementary information

Proteolysis inhibition by hibernating bear serum leads to increased protein content in human muscle cells

Stéphanie Chanon[1], Blandine Chazarin[2,3,4], Benoit Toubhans[1], Christine Durand[1], Isabelle Chery[2,5], Maud Robert[1,6], Aurélie Vieille-Marchiset[1], Jon E. Swenson[7,8], Andreas Zedrosser[9,10], Alina L. Evans[11], Sven Brunberg[7], Jon M. Arnemo[11,12], Guillemette Gauquelin-Koch[4], Kenneth B Storey[13], Chantal Simon[1], Stéphane Blanc[2,5], Fabrice Bertile[2,3], Etienne Lefai*[1]

Please find below the original images of all western blot and gel staining included in our article.

All image came from Chemidoc Biorad device, files were directly exported without modifications in tiff format. For figure 4, acquisition of the chemiluminescence of two different membranes was perfomed at the same time. Selection (dashed lines) indicate the part of the image that has been used to built the figures.

Figure 2

IB: anti puromycin

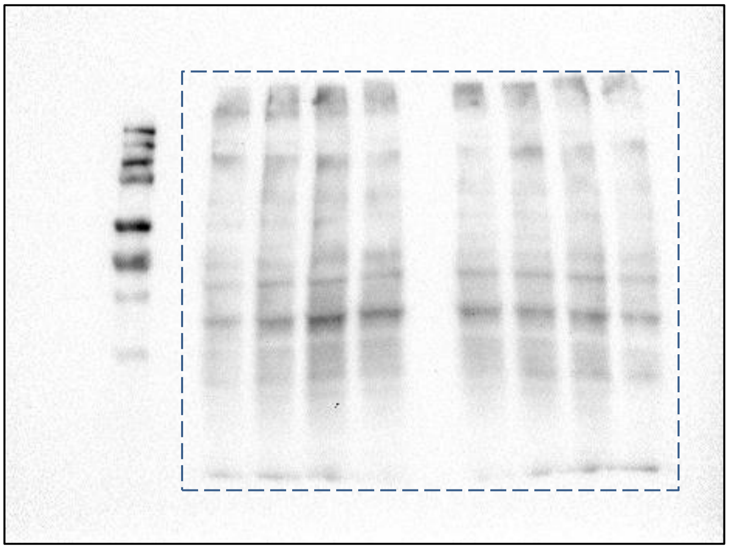

IB: tubulin

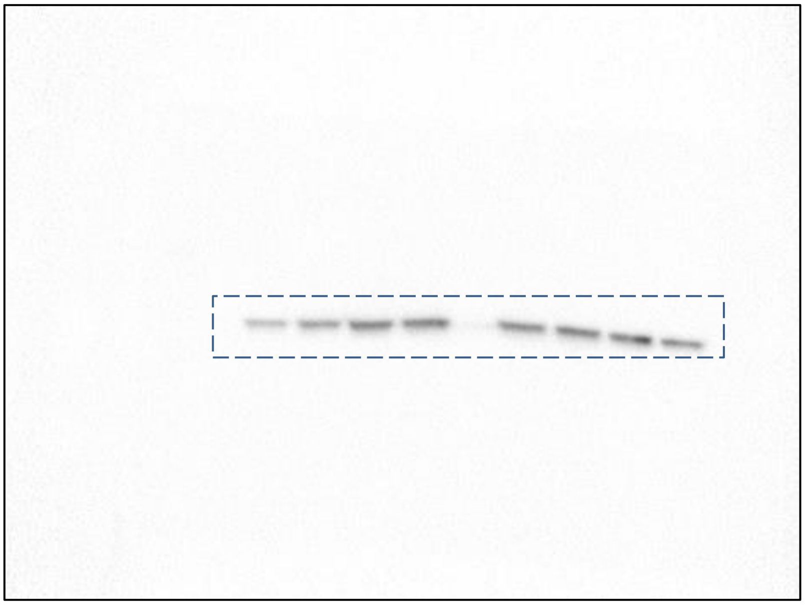

Figure 3

IB: anti ubiquitin

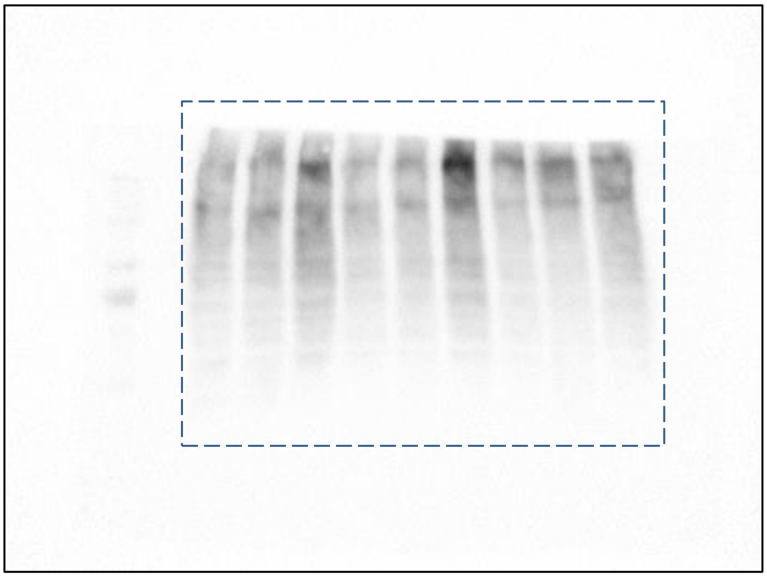

TGX staining

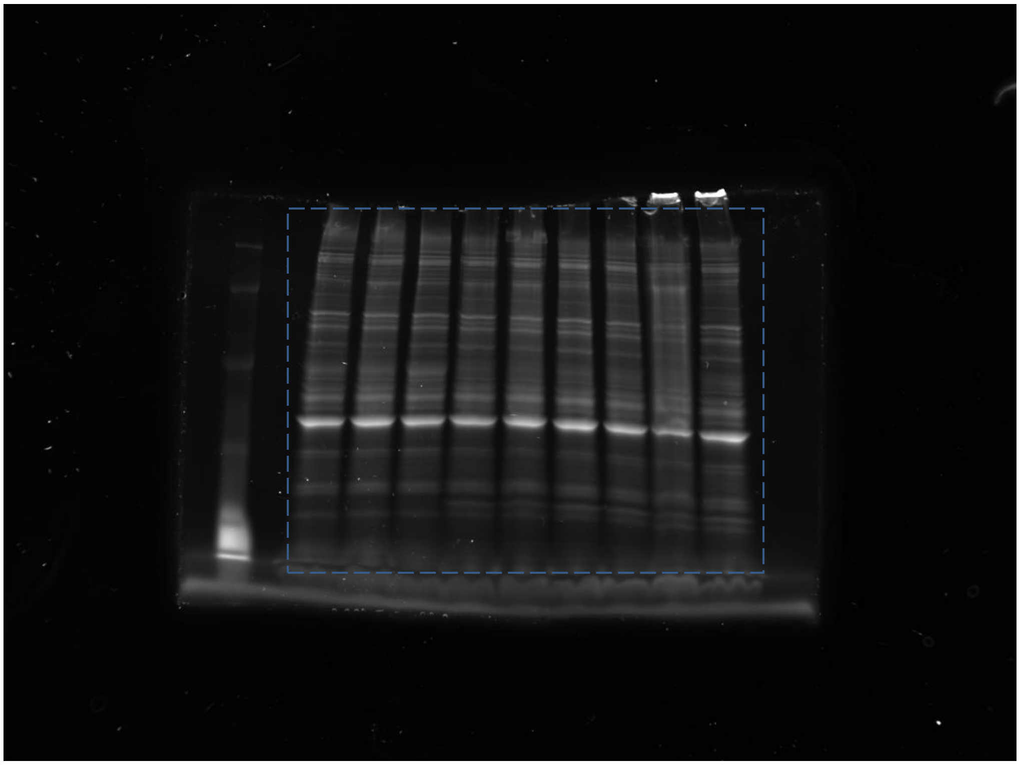

Figure 4

SQSTM1

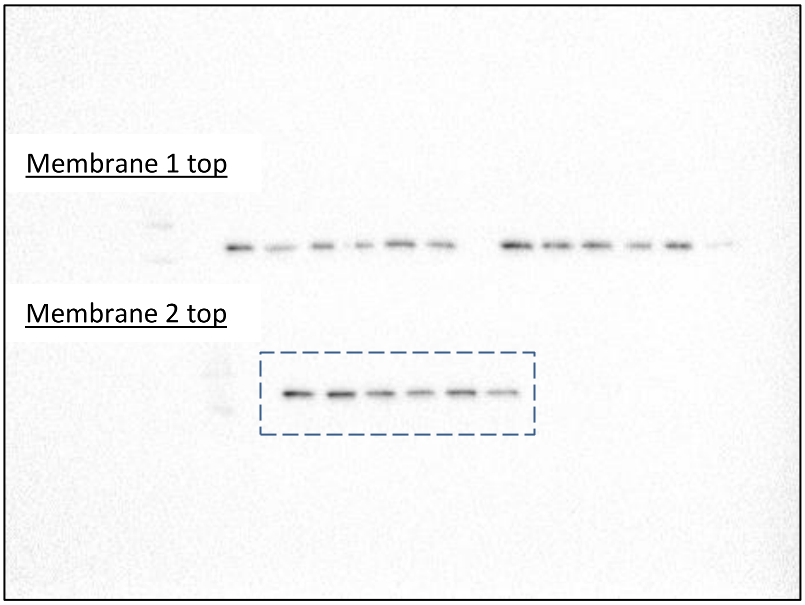

Tubulin

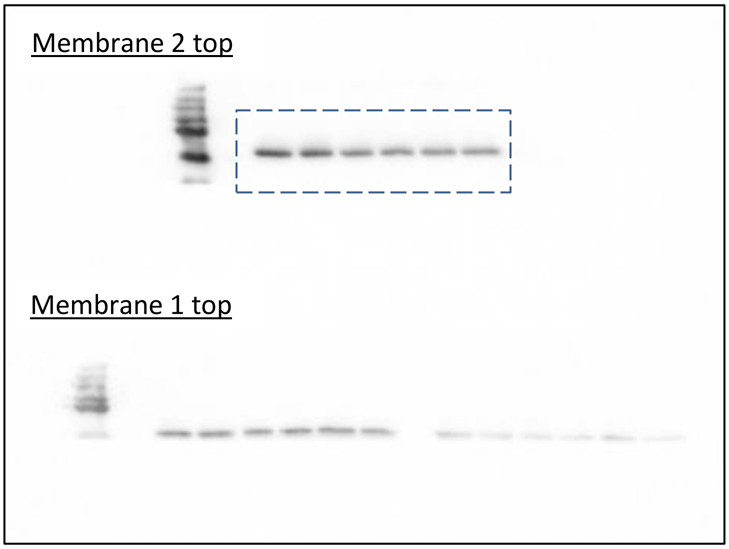

LC3bI et LC3bII

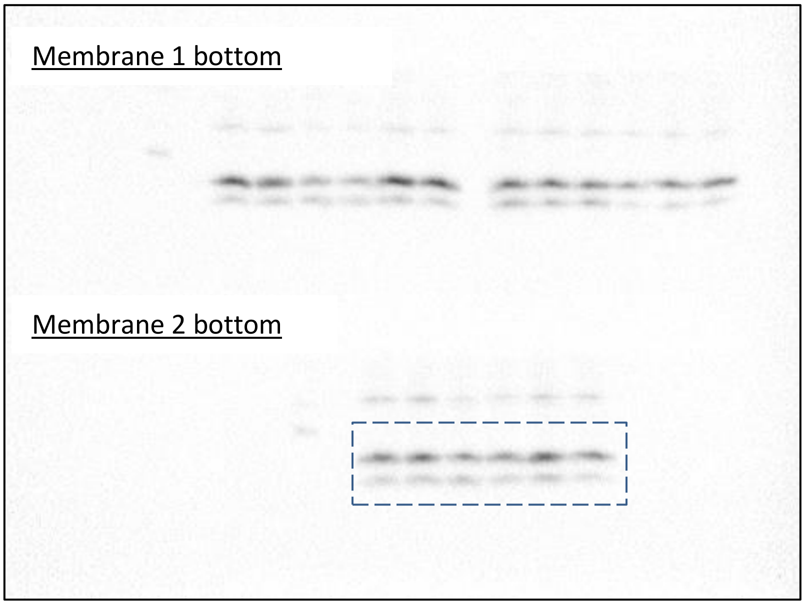

Figure 5

P-mTOR

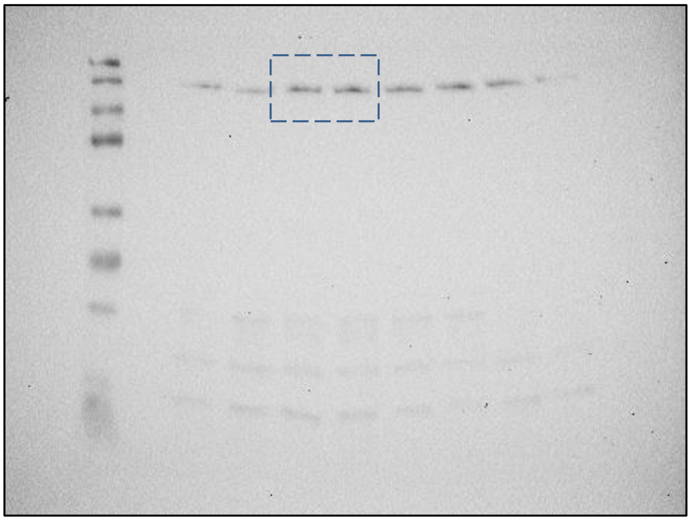

mTOR

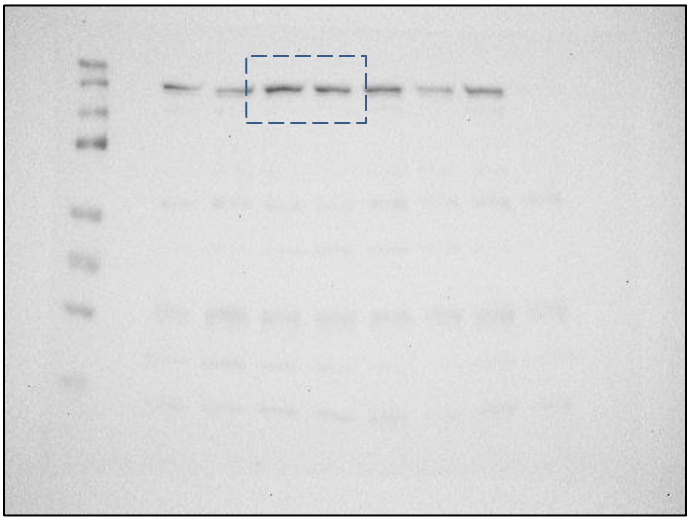

P-PKB

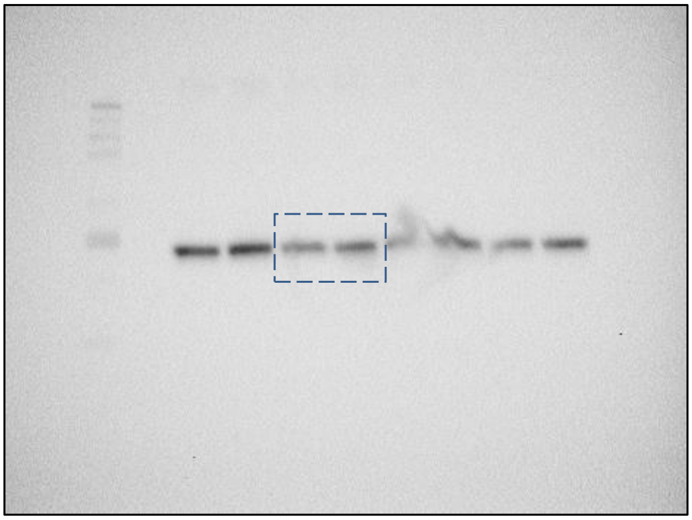

PKB

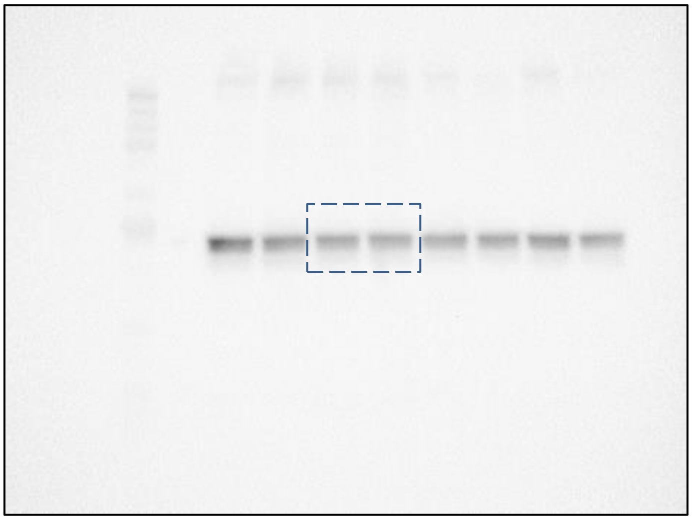

P-FOXO3

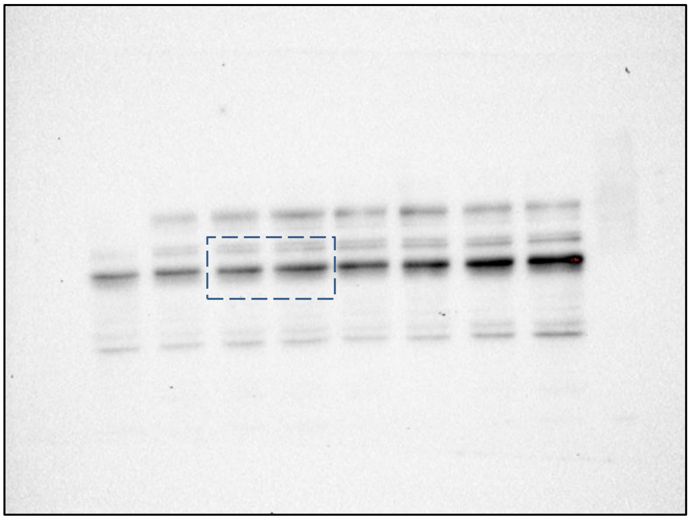

Tubulin

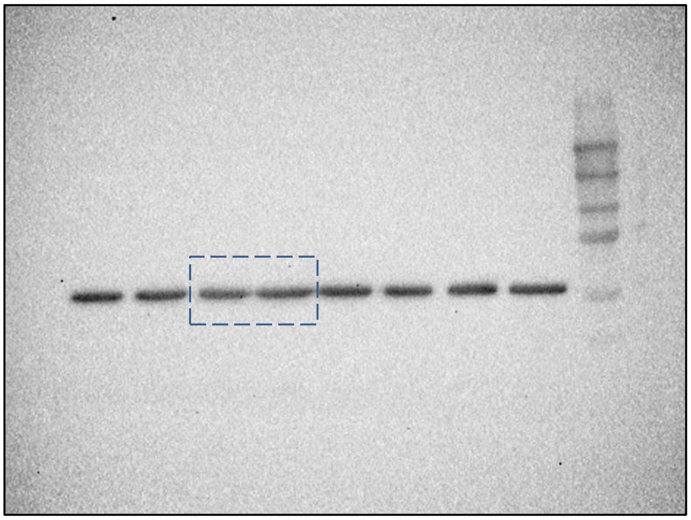

P-S6k

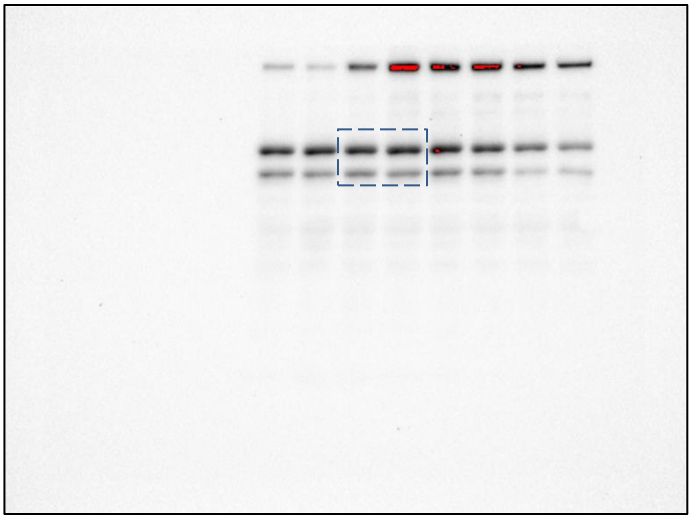

S6k

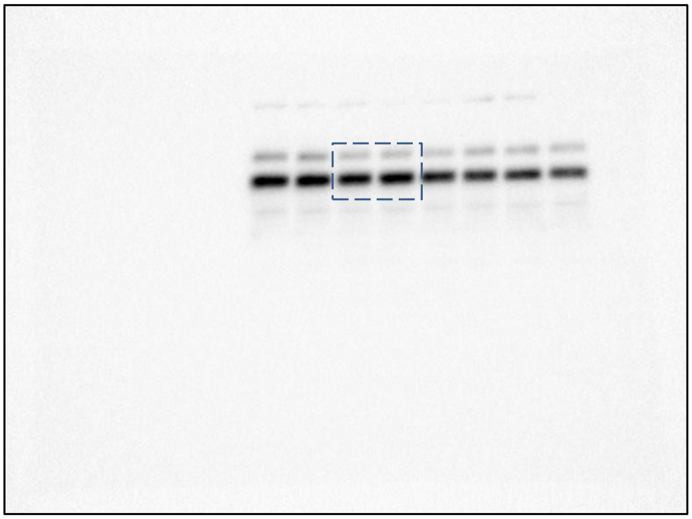

P-GSK3

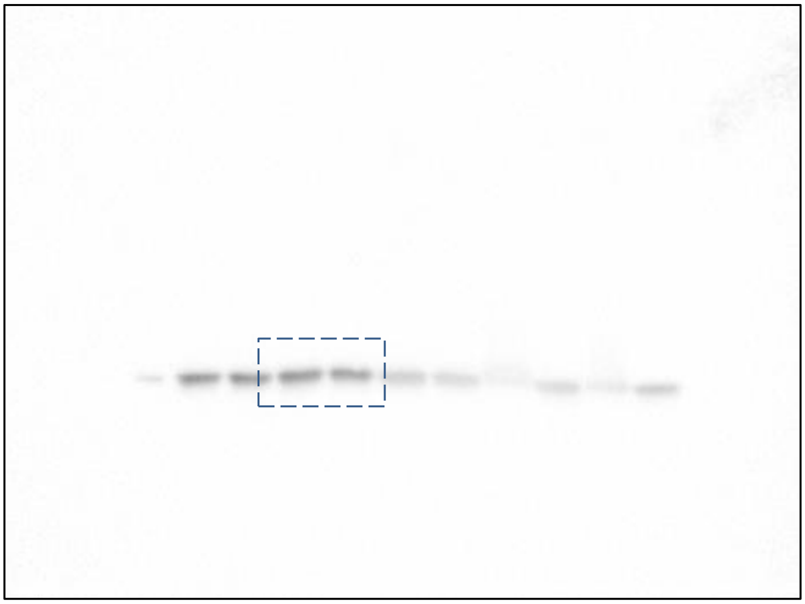

GSK3

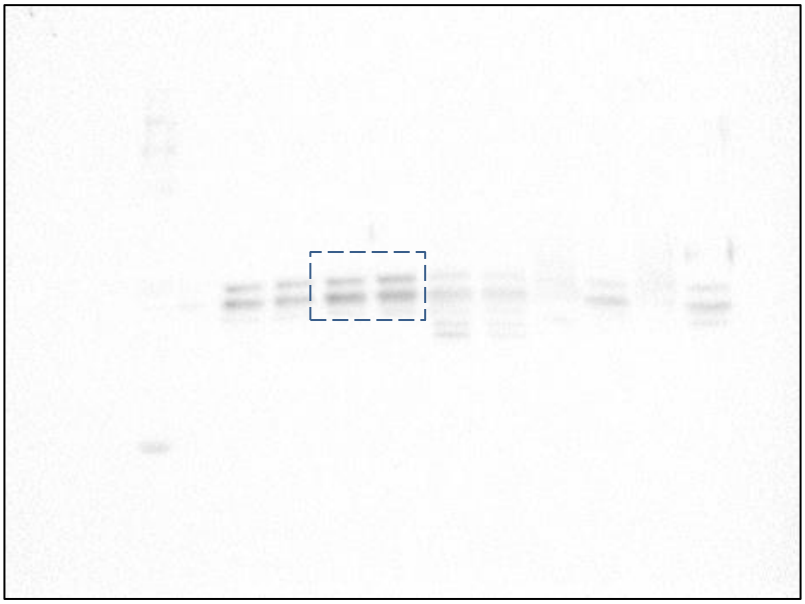

P-SGK

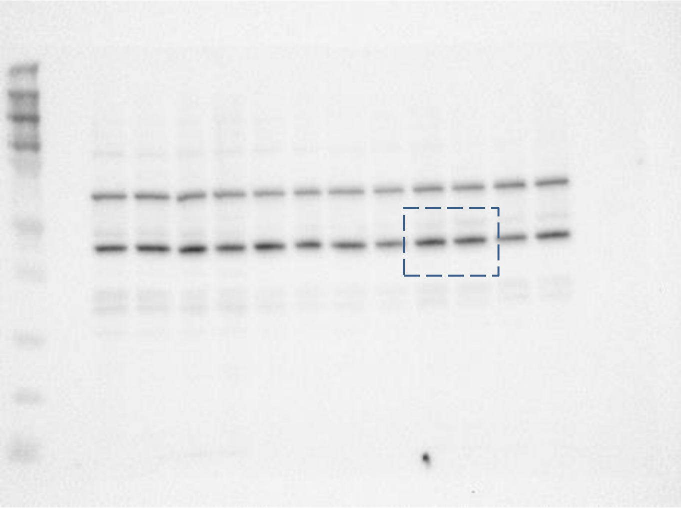

SGK

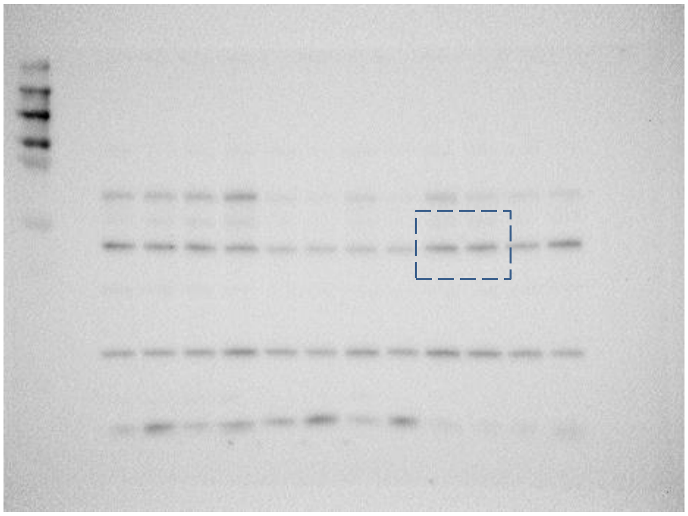

Supplement: Supplementary file 2 — Supplementary Information [file 41598_2018_23891_MOESM2_ESM.pdf]
